# p21^CIP/WAF1^ saRNA inhibits proliferative vitreoretinopathy in a rabbit model

**DOI:** 10.1371/journal.pone.0282063

**Published:** 2023-02-23

**Authors:** Qi Zhang, Yangchen Guo, Moorim Kang, Wei-Hsiang Lin, Jian-Cheng Wu, Ying Yu, Long-Cheng Li, Aimin Sang

**Affiliations:** 1 Department of Ophthalmology, Affiliated Hospital and Medical School of Nantong University, Nantong City, Jiangsu Province, China; 2 Dalian Medical University, Lvshunkou District, Dalian City, Liaoning Province, China; 3 Nantong University, Nantong City, Jiangsu Province, China; 4 Ractigen Therapeutics, Nantong City, Jiangsu Province, China; 5 Institute of Reproductive Medicine, Nantong University, Nantong City, Jiangsu Province, China; Charite Universitatsmedizin Berlin, GERMANY

## Abstract

**Purpose:**

Proliferative vitreoretinopathy (PVR) is a disease process resulting from proliferation of retinal pigment epithelial (RPE) cells in the vitreous and periretinal area, leading to periretinal membrane formation and traction and eventually to postoperative failure after vitreo-retinal surgery for primary rhegmatogenous retinal detachment (RRD). The present study was designed to test the therapeutic potential of a p21^CIP/WAF1^ (p21) inducing saRNA for PVR.

**Methods:**

A chemically modified p21 saRNA (RAG1-40-53) was tested in cultured human RPE cells for p21 induction and for the inhibition of cell proliferation, migration and cell cycle progression. RAG1-40-53 was further conjugated to a cholesterol moiety and tested for pharmacokinetics and pharmacodynamics in rabbit eyes and for therapeutic effects after intravitreal administration in a rabbit PVR model established by injecting human RPE cells.

**Results:**

RAG1-40-53 (0.3 mg, 1 mg) significantly induced p21 expression in RPE cells and inhibited cell proliferation, the progression of cell cycle at the G0/G1 phase and TGF-β1 induced migration. After a single intravitreal injection into rabbit eyes, cholesterol-conjugated RAG1-40-53 exhibited sustained concentration in the vitreal humor beyond at least 8 days and prevented the progression of established PVR.

**Conclusion:**

p21 saRNA could represent a novel therapeutics for PVR by exerting a antiproliferation and antimigration effect on RPE cells.

## Introduction

PVR is a disease process that follows the proliferation of ectopic cell sheets in the vitreous and/or periretinal area, causing periretinal membrane formation and traction, in patients with rhegmatogenous retinal detachment (RRD) and is the major cause for postoperative failure after vitreo-retinal surgery for primary RRD. PVR occurs in 5%–10% of all RRD and is implicated in redetachment after surgery in 75% of cases, representing a major barrier to successful repair of RD [1].

RRD causes a breakdown in the blood–retinal barrier (BRB), leading to cell migration and proliferation, with the cells involved mainly being the retinal pigment epithelial (RPE) cells, fibrous astrocytes, fibroblasts, myofibroblasts, and macrophages [2]. This process also attracts by chemotaxis a number of inflammatory cells involved in the healing process like macrophages, lymphocytes, and polymorphonuclear cells [3].

Different signals have been found to underlie the migration and proliferation of RPE cells, including loss of contact, response to factors present in the vitreous and secretions by inflammatory cells [4].

Effective treatment of PVR remains as a clinical challenge in ophthalmology. Although modern vitreoretinal microsurgery has obtained satisfactory results in the treatment of PVR, operational complications and/ or poor visual acuity have been associated outcomes in many cases [5]. Anti-inflammatory compounds, antiproliferative agents, and antigrowth factor inhibitors have been identified as major candidate drugs for the pharmacological suppression of PVR. However, their poor efficacy and multiple side effects limit their use in adjunctive therapy [5]. Therefore, it is important to develop new strategies for the prevention and treatment of PVR.

p21^WAF1/CIP1^ (p21) protein encoded by the gene CDKN1A (cyclin-dependent kinase inhibitor 1A) is a well-defined cyclin-dependent kinase inhibitor [6] that belongs to the Cip/Kip family of cyclin-dependent kinase inhibitors and is a key mediator in various cellular processes such as cell death, DNA repair, and senescence [7]. p21 overexpression causes G1- and G2- or S-phase arrest [8]. Transcriptional regulation mediated by various transcription factors (p53, Sp1/Sp3, and c-Myc) plays a key role in the expression and activity of p21 [9]. p21 is a gene that inhibits cell proliferation, although loss-of-function mutations in p21 are rare. Therefore, p21 is an ideal target for RNA-mediated inhibition of cell growth.

RNA activation (RNAa) is a gene regulation mechanism by which promoter-targeted small activating RNAs (saRNAs) induce transcriptional gene activation in a sequence specific manner [6, 10]. Targeted activation of therapeutic genes by saRNAs has been demonstrated in a variety of disease models including cancer and noncancerous diseases [11, 12]. A saRNA drug designed to activate the tumor suppressor gene CEBPA is in phase II trial for the treatment of liver cancer [12].

saRNA-guided p21 activation has been shown to elicit an anti-proliferative effect on various tumor cells, as well as benign cells and to exhibit antitumor role in animal models of cancer [6, 13, 14]. Previous studies have shown that overexpression of rabbit p21 via adenovirus vector has an inhibitory effect on the development of PVR in a rabbit PVR model [8]. Furthermore, adenovirus vector-mediated delivery of p21 inhibited retinal neovascularization (RNV) in a rat model of oxygen-induced retinopathy (OIR) [15]. These studies suggest that p21 could be a therapeutic target of saRNA for the prevention or treatment of PVR.

In the present study, a p21 saRNA (RAG1-40-53) was developed and tested for antiproliferation and anti-migration effect in cultured RPE cells and its efficacy in inhibiting the development of PVR in a PVR model established in rabbit eyes by intravitreal injection of human RPE-19 cells.

## Materials and methods

### Duplex RNAs

Chemically modified duplex saRNA and control duplex were synthesized by Ractigen Therapeutics. They contain an optimized confidential pattern of modifications including 2’fluoro, 2’ O-methyl and backbone phosphothioate replacement. The sequence used are listed in Table 1.

**Table 1:** Sequences of oligonucleotides and PCR primers.

Duplex name	Sense	Antisense
RAG1-40-53	CCAACUCAUUCUCCAAGUC	UACUUGGAGAAUGAGUUGGCA
dsCon2	ACUACUGAGUGACAGUAGATT	UCUACUGUCACUCAGUAGUTT
RAG1-40-53-C1	Chol-TEG-CCAACUCAUUCUCCAAGUC	UACUUGGAGAAUGAGUUGGCA
RAG1-40-53-Scr	Chol-TEG-CUAGCUCGUUCUUCCAUGC	UCAUGGAAGAACGAGCUAGCA
Primer name	Forward	Reverse
p21	GGA AGA CCA TGT GGA CCT GT	GGA TTA GGG CTT CCT CTT GG
GAPDH	ATC ACC ATC TTC CAG GAG CGA	TTC TCC ATG GTG AAG ACG
Stem-loop	CGG GCT ACT TGG AGA ATG AGT TG	GTG CAG GGT CCG AGG T
RT primer	GTC GTA TCC AGT GCA GGG TCC GAG GTA TTC GCA CTG GAT ACG ACA GTG CC	

Note: Chol: cholesterol; TEG: triethylene glycol

### Cell culture

ARPE-19 cell line (human RPE cells) was purchased from Shanghai Enzyme Research Biotechnology Co., LTD. Human RPE cells (ARPE-19 cell line) were cultured in DMEM /F12 medium comprising 10% fetal bovine serum (FBS; Hyclone, USA), 100 U/mL penicillin, and 100 μg/mL streptomycin under 5% CO_2_ atmosphere at 37°C. Small activated RNA was transfected with RNAiMax (Invitrogen, Carlsbad, CA) at a concentration of 10 nM (unless otherwise specified) according to manufacturer’s instructions. The transfected cells were be used for subsequent in vitro experiments.

### RNA isolation and reverse transcription quantitative polymerase chain reaction (RT-qPCR)

Total cellular RNA was extracted from cultured cells using the RNeasy Plus Mini kit (Qiagen) according to its instructions. RNA (1 μg) was reversely transcribed into cDNA using PrimeScript RT kit containing gDNA Eraser (Takara, Shlga, Japan). RT-qPCR was conducted using p21 specific primers and SYBR Premix Ex Taq II (Takara, Shlga, Japan) reagent in ABI 7500 Fast Real-Time PCR System (Applied Biosystems). The reaction conditions were as follows: 95°C for 5 seconds, 60°C for 20 seconds, 72°C for 10 seconds, 40 cycles were amplified. GAPDH was also amplified as an internal control for RNA loading. All primer sequences are listed in Table 1.

### Cell proliferation assay

Cells were seeded in a 96-well plate with 2–4×10^3^ cells/well and transfected the next day. Three days after transfection, number of viable cells was detected using the CCK8 kit (Dojindo) according to its instructions. Briefly, at the end of the transfection, 10 μl of CCK8 solution was added to each well and incubate at 37°C for 1 hour before measuring the absorbance value at 450 nm with a microplate reader.

### Scratch wound healing assay

ARPE-19 cells were grown in 12-well plates with 2×10^5^ cell/well and then transfected with duplex RNA using RNAiMAX. On the second day, first use marker behind the 12-hole plate and draw horizontal lines evenly with a ruler. Then use 1 ml blue spear head to compare the ruler and try to hang the horizontal line scratches on the back. The spear head should be vertical and not incline. The cells were washed with PBS for 3 times, the subtracted cells were removed, and serum-free culture or serum-free medium containing 10 ng/ml TGF- B was added. Put it into a 37°C 5% CO2 incubator for cultivation. Samples were taken at 0, 24, 48 and 72 hours, and each scratch was divided into three holes for photographing. Image J software was used to calculate the mean distance between cells.

### Cell cycle assay

RPE cells (1 × 10^5^/well) were seeded in a 12-well plate and transfected with duplex RNA for 48 h using RNAiMAX. After transfection, cells were trypsinized and centrifuged at 3000 rpm. The resulted cell pallet was resuspended in PBS to give a cell concentration of 2 × 10^5^ cells/0.2 ml. Subsequently, the cells were harvested and add 70% ice-cold ethanol overnight incubation at 4°C. Before analysis, the cells were washed with PBS and centrifuge less than 3000 rpm in EP tube twice. Krishan buffer (200 μL) and incubate were then added to suspend cells for 1 h at 4°C. DNA histograms were obtained using the Becton-Dickenson FACS Vantage flow cytometry system (Becton-Dickenson, USA), and Cell Quest software version 3.2 (Becton-Dickenson, USA) was used for cell cycle distribution and apoptosis analyses. Experiments were performed in two replicates and repeated twice.

### Apoptosis analysis

Human RPE cells (1 × 10^5^) were incubated in a 12-well plate with Mock, RAG1-40-53, and dsCon2 groups of transfection reagents (RNAiMAX: 3 μL/well) for 48 h. Then cells were trypsinized (100 μL trypsin for 2 min) and centrifuged less than 3000 rpm in EP tube to harvest cells. Count and make the cell numbers on 2 × 10^5^ cells/0.2 ml. Add PBS and centrifuge less than 3000 rpm in EP tube twice. For Staining cells add 5 μL of FITC Annexin V and 5 μL PI and 100 μL 1× Binding Buffer mixture, and incubate for 15 min at RT in the dark. Add 100 μL of 1× Binding Buffer to each tube, transfer cells to U-shaped 96-well plate, analyze by flow cytometry within 1 h.

### Rabbit PVR model creation and treatment by intravitreal administration of saRNA

All animal experiments in this study were conducted in accordance with the Animal Ethics Committee of Nantong University. Statement requirements for ARVO (Association for Research in Vision and Ophthalmology). The study was approved by Laboratory Animal Center of Nantong University (Protocol No. S20220603-001).

30 adult female New Zealand white rabbits (weight: 1.8–2.2 kg; age 5–6 months) were purchased from Laboratory Animal Center of Nantong University. All the rabbits were anesthetized with an intramuscular injection of ketamine (50 mg/kg) and promethazine (25 mg/kg). Before surgery, the pupils of the rabbits were dilated with tropicamide, and antibiotics were administered to the rabbits. PVR was induced through an intravitreal injection of 2.5 × 10^5^ human ARPE-19 cells in 0.1 mL PRP with a 30-gauge needle. An anterior chamber paracentesis was made and approximately 0.1 mL aqueous humor was drained with a 30-gauge needle before the injection. Fundus images were captured on day 7, day 14, and day 21.

Fifteen days after PVR induction, based on previous drug dosage studies, the rabbits were randomly divided into 5 groups: PBS group (n = 4, intravitreal injection of 100 μL PBS); Methotrexate group (n = 4, vitreous injection of 100 μL methotrexate); RAG1-40-53-C1 group (n = 12, vitreous injection of 100 μL RAG1-40-53-C1 [0.1 mg/0.3 mg/1.0 mg]) (Table 2). MTX, RAG1-40-53-C1, or PBS was injected into the posterior superior temporal region of the lens under the guidance of an operating microscope, and a glass syringe (Hamilton Co., Reynolds, NV) was connected to a metal needle to avoid penetrating the lens or vortex vein injury. The injection site was located at the heterochromatic margin 3 mm above the posterior temporal.

**Table 2:** Treatment groups and outcome assessed by Fastenberg’s classification.

Group	Treatment	Dose/vol	# of treated eyes	Pre-dosing Fastenberg’s score	Post-dosing Fastenberg’s score
PBS	PBS	0.02mg/100μl	4	3	5
MTX	methotrexate	0.4mg/100μl	4	4	2
RAG1-40-53-C1 (0.1 mg)	RAG1-40-53-C1	0.1 mg/100 μl	4	5	4
RAG1-40-53-C1 (0.3 mg)	RAG1-40-53-C1	0.3 mg/100 μl	4	4	3
RAG1-40-53-C1 (1 mg)	RAG1-40-53-C1	1 mg/100 μl	4	5	2

On day 22, all rabbits were administered inhalation anesthesia, and fundus conditions were observed through fundus photography and optical coherence tomography (OCT). The same angle was considered to observe fundus conditions of the rabbits before and after modeling, and the formation of the fibrous membrane around the macula was observed through OCT. PVR changes were graded by using Fastenberg’s classification [16] with normal eye being stage 0, and most sever PVR (total retinal detachment) being stage 5. Grade 0 had no visible pathology; Grade 1 had the presence of an epiretinal membrane; Grade 2 had epiretinal membrane with focal retinal traction; Grade 3 had localized detachment of medullary rays; Grade 4 had extensive retinal detachment (total detachment of medullary ray and peripapillary retina) and Grade 5 had total retinal detachment with fixed retinal folds. At the end of the experiment, the rabbits were sacrificed by administering anesthetic overdose

### Pharmacokinetics and pharmacodynamics of RAG1-40-53-C1

Fifteen rabbits were divided into three groups to receive intravitreal of saline, RAG1-40-53-C1 0.1 mg and RAG1-40-53-C1 0.3 mg (50 μl/eye), respectively. On day 0, day 2, day 4, day 6 and day 8, ***vitreous*** humor from vitreous body, and plasma were collected and analyzed for oligonucleotide concentration by stem-loop qPCR.

To assess oligonucleotide concentration in vitreous humor and plasma, stem-loop qPCR was performed. Briefly, a standard curve was established for vitreous humor and plasma by spiking known amount of RAG1-40-53. Experimental and spiked samples were reverse transcribed into RNA by adding Reagent (RR037A, Takara) and then amplified by qPCR using Forward primer (10 uM), Reverse primer (10 uM), RT product (1:40). The reaction conditions were as follows: 95°C for 5 seconds, 60°C for 20 seconds, 72°C for 10 seconds, 40 cycles were amplified. The sequence of the primers are listed in Table 1.

For assessing pharmacodynamics (PD), RAG1-40-53-C1 was mixed with ARPE-19 cells at concentrations of 0.1, 0.3, and 1 mg/ml or a scrambled duplex (RAG1-40-53-Scr) at 1 mg/ml and immediately injected into rabbit eyes. After 3 days, the rabbit eye was dissected and cells were recovered from the vitreous humor by centrifuge to assess the expression level of p21 by RT-qPCR.

### Statistical analysis

Results are expressed as the mean. One-way ANOVA followed by Dunnett’s t-test was used for statistical analysis using GraphPad Prism (GraphPad Software, La Jolla, CA, USA). A *P* value of <0.05 was considered statistically significant.

## Results

### RAG1-40-53 induces p21 expression and cytostasis in ARPE-19 cells

**Fig 1:**
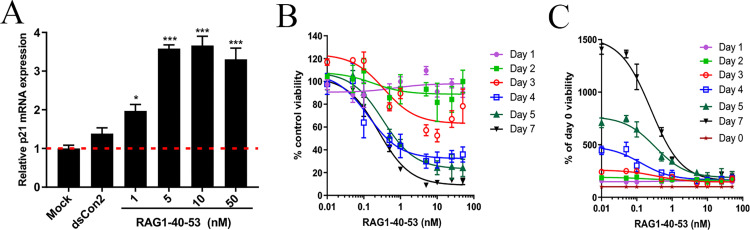
RAG1-40-53 induced p21 mRNA expression and caused a cytostatic effect in ARPE-19 cells. (A) ARPE-19 cells were transfected with the indicated duplex for 72 hours and mRNA expression of p21 was evaluated by RT-qPCR after RNA isolation from the treated cells and reverse transcription to convert the RNA into cDNA. (B) ARPE-19 cells were transfected with RAG1-40-53 at the indicated concentrations (ranging from 0.01, 0.05, 0.1, 0.5, 1,5, 10, 25, to 50 nM) or mock transfected for a duration ranging from 1 to 7 days. Viable cells were counted by using a CCK-8 kit and plotted as % of control treatment (B) and % of before transfection (day 0) (C). Error bars represent the mean SEM of two independent experiments. *P < 0.05, ***P < 0.001.

To induce p21 expression in ARPE-19 cells, a p21 promoter-targeting saRNA (RAG1-40-53) with chemical modification was transfected in the cells at different concentrations for 72 h. As shown in Fig 1A, compared to mock transfection, RAG1-40-53 at 1, 5 and 10 nM caused an 1.97, 3.8 and 3.66 fold induction of p21 mRNA respectively, while a higher concentration (50 nM) did not further increase p21 expression (3.30 fold). To evaluate potential antiproliferative impact of p21 activation by RAG1-40-53 on ARPE-19 cells, viable cells were counted by CCK-8 assay after transfecting RAG1-40-53 at different concentrations ranging from 0.01 nM to 50 nM for a duration ranging from 1 day to 7 days. As shown in Fig 1B, a dose and time-dependent inhibition of cell viability was obvious with an extrapolated IC_50_ of 0.36 nM at day 3. To further determine whether the decrease in viability was due to cytostatic or cytotoxic effect, the viability data was plotted as percentage of viable cells relative to day 0 (before treatment). As shown in Fig 1C, treatment of ARPE-19 cells by RAG1-40-53 at all concentrations did not cause a net decrease in the number of viable cells compared to that of pre-treatment, indicating that RAG1-40-53 elicited a cytostatic instead of cytostatic effect on ARPE-19 cells, which is an important safety property for intravitreal treatment.

### RAG1-40-53 inhibited cell cycle progression of ARPE-19 cells

**Fig 2:**
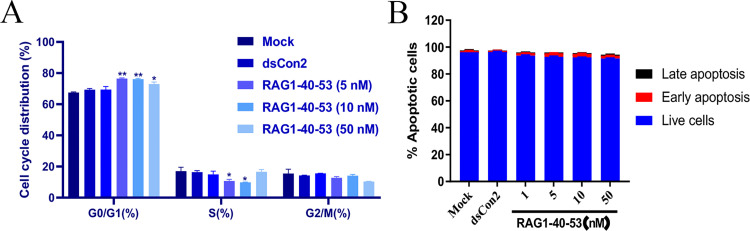
RAG1-40-53(saRNA) alleviated cell cycle and cell toxic in ARPE-19 cells. (A) Flow cytometry cycle of the control group and the transfection group with different concentrations of saRNA. Bar graph represents the quantitative data of RPE cell cycle distribution in Mock, dsCon2 and RAG1-40-53(1nM, 5nM, 10nM, 50nM) groups. Each value represents the mean ± SD of two independent experiments. Each performed in copies. The saRNA transfection group was significantly different from the control group (*P < 0.05, **P<0.001). (B) Apoptosis analysis was used to determine whether saRNA (RAG1-40-53) was toxic to ARPE-19 cells. There was no significant difference in cytotoxicity between the transfected saRNA group and the control group. (P>0.05).

To determine the pharmacological mechanism for the observed antiproliferative effect of RAG1-40-53, cell cycle profile was assessed in ARPE-19 cells by flow cytometry after PI staining. As shown in Fig 2A, compared with the control treatments, RAG1-40-53 treated cells showed a statistically significant increase in the G0/G1 population and an concurrent decrease in the S population (*P* < 0.05). The results indicated that the RAG1-40-53 arrests the cell cycle of ARPE-19 cells at the G0/G1 phase.

To evaluate *in vitro* safety and further support the finding that RAG1-40-53 mainly induced a cytostatic instead of a cytotoxic effect, apoptosis was evaluated by flow cytometry after Annexin V and PI staining. As shown in Fig 2C, treatment with RAG1-40-53 at different concentrations (1, 5, 10, and 50 nM) showed no significant change in the proportion of apoptotic cells, indicating that RAG1-40-53 neither caused toxicity to ARPE-19 cells nor induced apoptosis.

### RAG1-40-53 inhibited TGF-β-induced ARPE-19 cell migration

**Fig 3:**
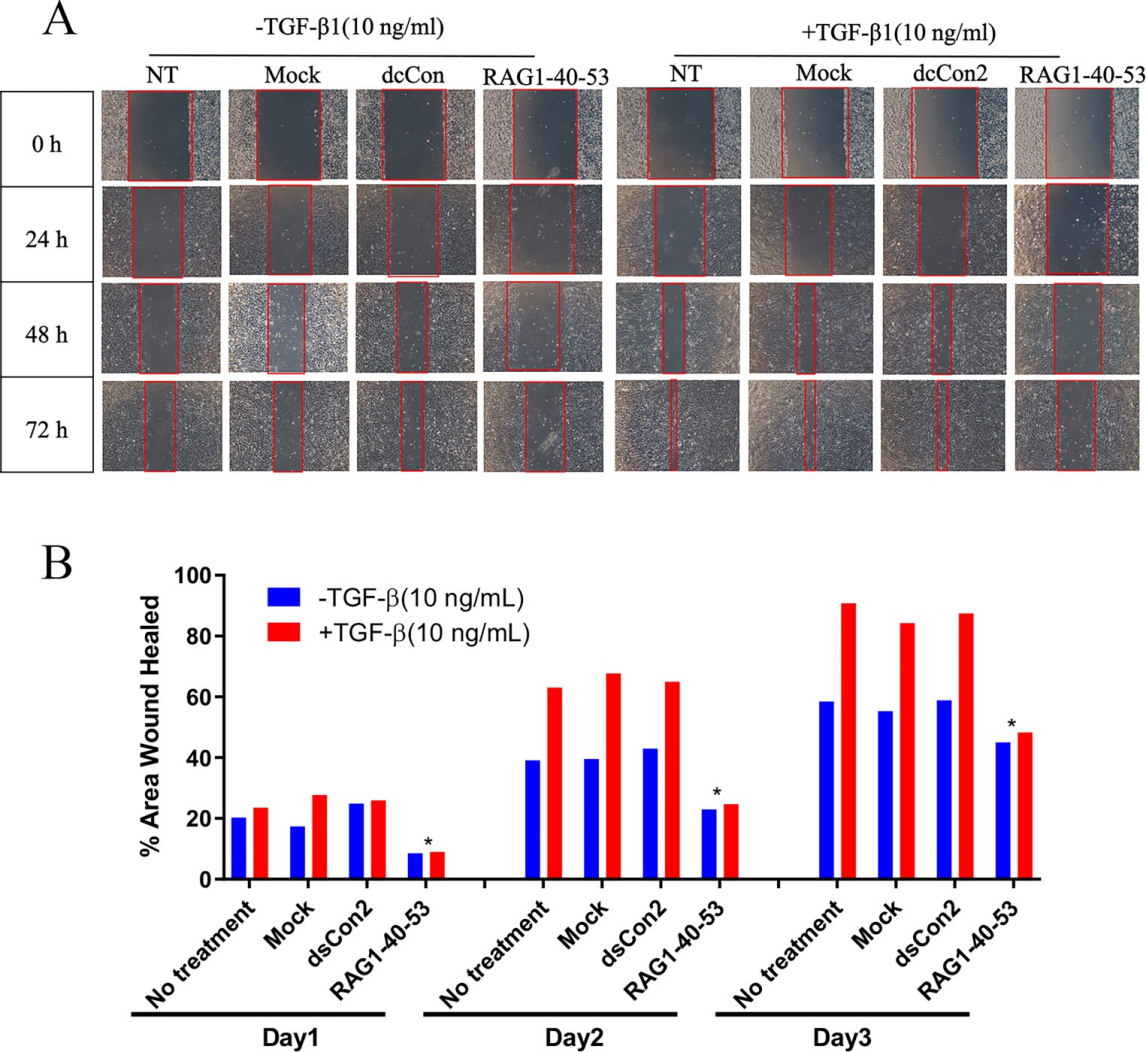
Effects of RAG1-40-53-C1 on ARPE-19 cell migration. (A) Scratch test, wound healing of ARPE-19 cells on 0h, 24h, 48h, 72h; (B) The area wound healed of the transfected saRNA group was significantly different from that of the three control groups.(* P < 0.05).

To determine whether p21 induction by RAG1-40-53 could have an effect on cell motility and migration, ARPE-19 cells were transfected with RAG1-40-53 in the presence and absence of TGF-β1 (10 ng/mL), a growth factor known to induce epithelial to mesenchymal transition (EMT), thereby promoting cell motility and invasiveness. As shown in Fig 3A and 3B, cells in control treatments (no treatment, mock and dsCon2) exhibited a decreased area of healed wound in the presence of TGF-β1, indicative of stimulated motility. However, compared to those control treatments, RAG1-40-53 (10 nM) treatment showed a significant inhibitory effect on cells’ motility at different time points (24, 48 and 72 h). This result suggests that p21 induction by RAG1-40-53 could suppress TGF-β1-induced EMT and motility which are an important pathogenic process of PVR.

### Cholesterol conjugated RAG1-40-53 inhibited ARPE-19 cell proliferation in free uptake treatment

**Fig 4:**
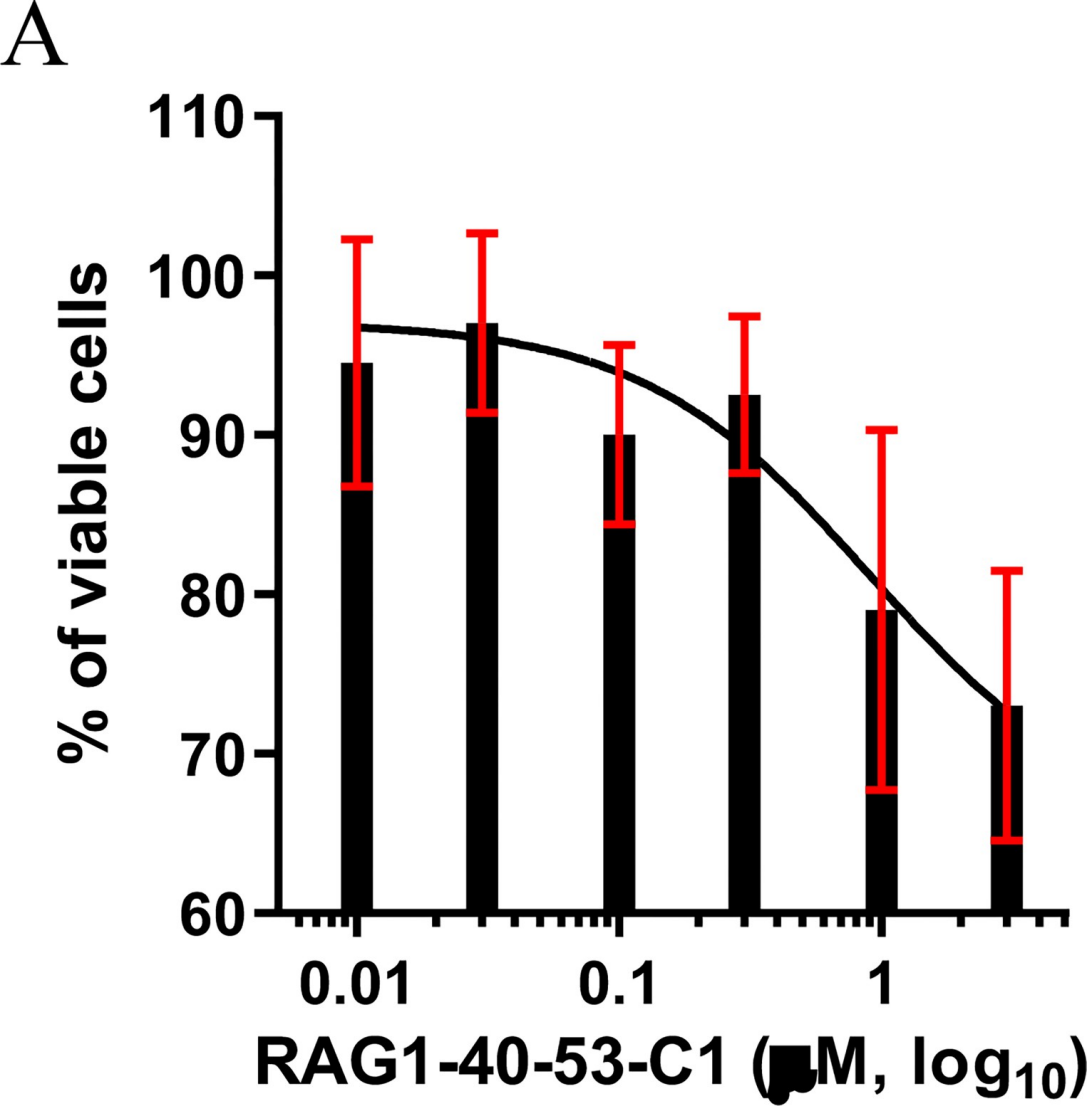
Cholesterol conjugated RAG1-40-53 inhibited ARPE-19 cell proliferation in free uptake treatment. RAG1-40-53-C1 treatment was found to reduce ARPE-19 cell viability in a dose-dependent manner with a calculated IC50 value at 0.9 μM and a 6x% maximum inhibition at 10 μM.

To be able to test *in vivo* therapeutic effects of RAG1-40-53, this duplex saRNA was conjugated to a cholesterol moiety via a linker to the 3’ end of its sense strand, resulting in RAG1-40-53-C1. The ARPE-19 cells by adding RAG1-40-53-C1 were analyzed for viability by CCK-8 assay. As shown in Fig 4, RAG1-40-53-C1 treatment was found to reduce ARPE-19 cell viability in a dose-dependent manner with a calculated IC_50_ value at 0.9 μM and a 27% maximum inhibition at 10 μM.

### Durable retention of intravitreal administrated RAG1-40-53-C1 in rabbit eyes with minimal systemic exposure

**Fig 5:**
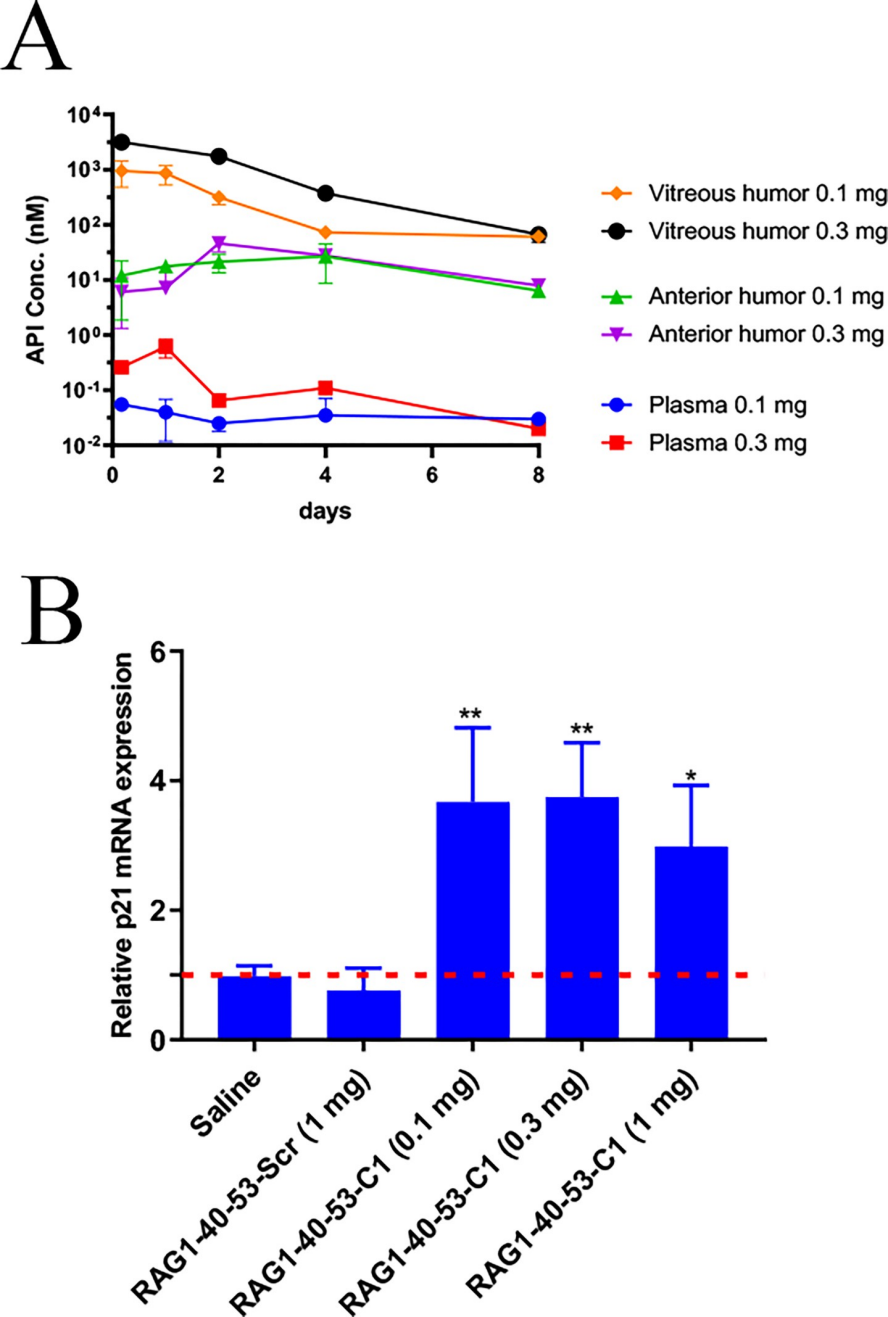
Durable retention of intravitreal administrated RAG1-40-53-C1 in rabbit eyes and minimal systemic exposure. (A) A standard curve was generated by spiking a known amount of RAG1-40-53-C1 into anterior humor, vitreous humor, and plasma samples from untreated rabbits. (B) Anterior humor, vitreous humor and plasma were collected on day 2, 4, 6 and 8 and the concentration of RAG1-40-53-C1 was determined by stem-loop qPCR. (*p<0.05; **p<0.01).

To determine the pharmacokinetics (PK) of intravitreal administrated RAG1-40-53-C1, 15 healthy New Zealand rabbits were divided into 3 groups with 5 in each group: Saline, RAG1-40-53-C1 low dose (0.1 mg), and RAG1-40-53-C1 high dose (0.3 mg). Saline or RAG1-40-53-C1 were injected into the vitreous space on day 0. Anterior humor, vitreous humor and plasma were collected on day 2, 4, 6 and 8 and the concentration of RAG1-40-53-C1 was determined by stem-loop qPCR. A standard curve was generated by spiking a known amount of RAG1-40-53-C1 into the anterior humor, vitreous humor, and plasma samples from untreated rabbits. As shown in Fig 5A, vitreous humor contained the highest and dose-dependent concentration of RAG1-40-53-C1 which declined slowly over a period of 8 days. RAG1-40-53-C1’s concentration in the anterior humor was significantly lower than that in the vitreous humor and remained very stable during the course of 8 days. In vitreous humor, RAG1-40-53-C1 exhibited a dose-dependent C_max_ at 4 h which were 1903.6 and 3149.8 nM for the 0.1 and 0.3 mg treated groups, respectively. In the plasma, the C_max_ of RAG1-40-53-C1 were 0.04 nM and 0.62 nM which were 0.002% and 0.019% of that of the vitreous humor. These data suggests that intravitreally administrated RAG1-40-53-C1 maintains durable concentration in the vitreous humor with minimal systemic exposure.

### RAG1-40-53-C1 induced p21 expression in rabbit eyes

To assess *in vivo* pharmacodynamic properties of RAG1-40-53-C1, ARPE-19 cells were mixed with RAG1-40-53-C1 and then injected rabbit vitreal body. p21 mRNA expression was assessed from the cells recovered 14 days from the injected rabbit eyes. As shown in Fig 5B, RAG1-40-53-C1 caused significant induction of p21 mRNA compared to either saline or control saRNA treatment.

### RAG1-40-53-C1 inhibited PVR progression in a rabbit PVR model

**Fig 6:**
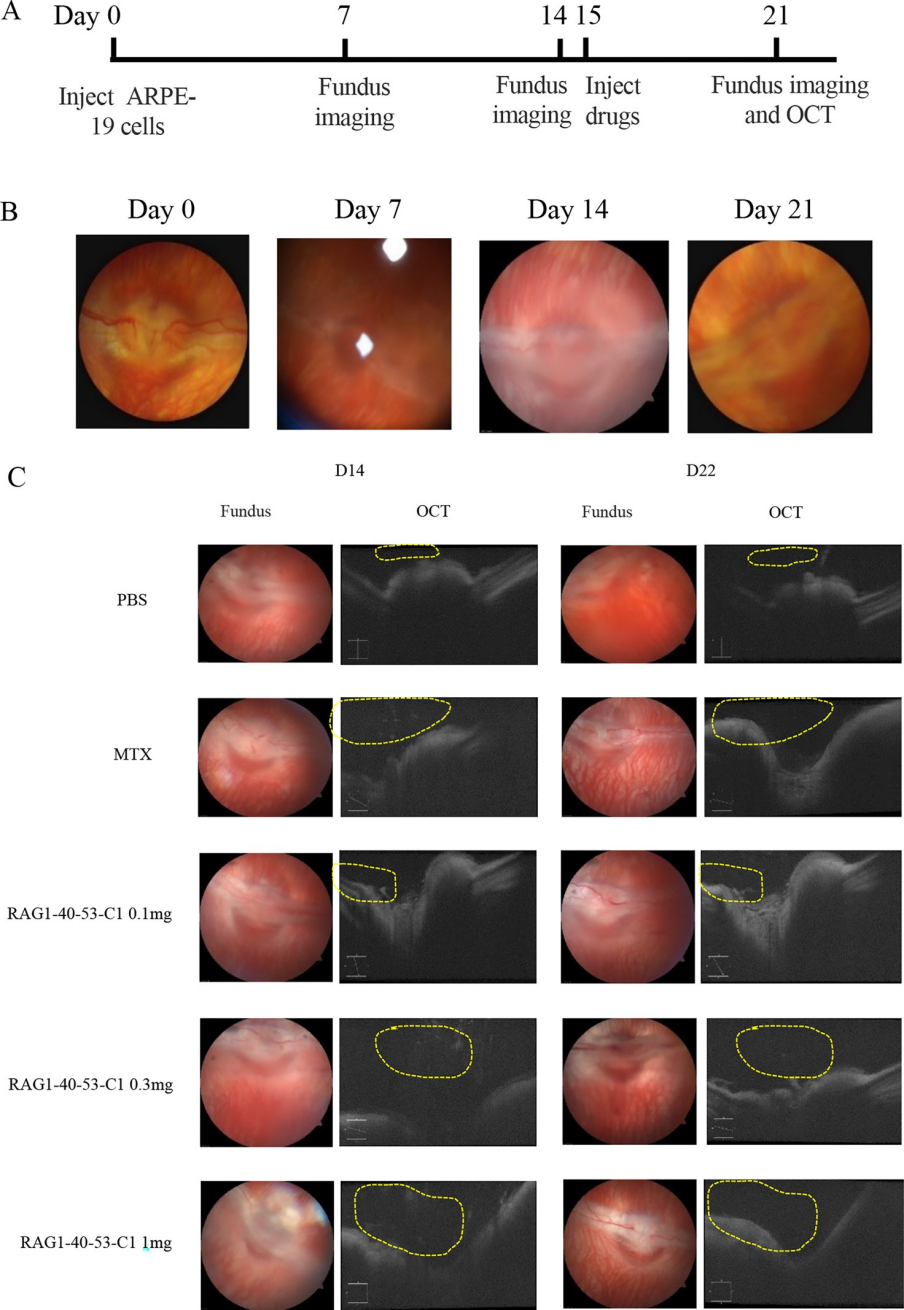
RAG1-40-53-C1(saRNA) inhibit the fibrosis of ARPE-19 cells and reduce the grade of PVR in vivo. (A) The PVR in vivo model was constructed in the eyes of rabbits, and the changes over time were observed by fundus photography. (B) Drug efficacy trial schedule. (C) Drug effect test. RAG1-40-53-C1 injection induced an increase in P21 mRNA expression in rabbit eyes. The yellow circles indicate fibrous new membrane like material. PBS group, MTX group, saRNA transfection group (RAG1-40-53-C1 0.1mg, RAG1-40-53-C1 0.3mg, RAG1-40-53-C1 1.0mg). The changes of PVR were observed by fundus photography and OCT on day 14 and 22. (*P < 0.05, **P < 0.01).

To test the efficacy of RAG1-40-53-C1 on inhibiting PVR development, PVR models were created in rabbit eyes by injecting 2.5 × 10^5^ ARPE-19 cells into the vitreous humor of the left eye of 26 healthy New Zealand white rabbits. On day 14, OCT and fundus imaging were taken to evaluate the formation of intraocular PVR (Fig 6B). Eyes with PVR score higher than 2 were randomly divided into 4 groups: Control (PBS), methotrexate, RAG1-40-53-C1 (0.1 mg), RAG1-40-53-C1 (0.3 mg), RAG1-40-53-C1 (1 mg) with 4 PVR model eyes in each group (Table 1) and received intravitreal treatment on day 15 (Fig 6A). Seven days post-treatment (day 22), OCT and fundus imaging were re-taken to evaluate the development of PVR.

As shown in Fig 6B, 7 days after cell injection, fundus photography in the PBS group showed turbidness in the vitreous cavity, suspected of having vitreous hemorrhage and white streaky material in the macula. Fundus photography in MTX group showed that after treatment, retinal flattening and blood vessels were normal without obvious dilation, tortuosity or bleeding.

Fundus photography in RAG1-40-53-C1 group showed that the anterior macular membrane was significantly improved with increased treatment doses. Retinal blood vessels were clearer than those in PBS group and no fundus hemorrhage was observed. On OCT examination as shown in area outlined by dotted yellow line in Fig 6C, eyes in the PBS group showed significant macular anterior membrane traction and edema, and thickening of the retinal neuroepithelium in the macular area. In contrast, eyes in the MTX and RAG1-40-53-C1 groups displayed significantly reduced proliferation of macular and surrounding fibrous membrane. These results suggest that treatment of PVR with RAG1-40-53-C1 significantly slowed the progression of PVR, and the therapeutic effect became more pronounced with increasing dose.

## Discussion

PVR is a common complication that occurs after corrective surgery for rhegmatogenous retinal detachment and there is no FDA approved pharmacological treatment for it. In the present study, the potential of RAG1-40-53, a saRNA designed to target the promoter of p21 gene to induce its expression at the transcriptional level, in inhibiting PVR development was tested in a rabbit model established by injecting human RPE cells and findings from this study supported the therapeutic efficacy of RAG1-40-53.

RPE cell migration and EMT are key processes in the development of PVR, and without these processes, RPE cells fail to transform into myofibroblast-like cells, gaining more energy to enter the vitreous lumen and settle on the retinal surface, thereby contributing to the formation of proliferating omental membranes [17]. In this in vitro study, RAG1-40-53 inhibited RPE cell migration by causing p21 overexpression. The migration of RPE cells depends on the phosphatidylinositol 3-kinase (PI3K)/protein kinase B (AKT)/mammalian target of rapamycin (mTOR) signaling pathway [18]. Additionally, cell cycle progression and survival induced by p21 depend on PI3K/AKT and c-Myc signaling pathway activation [19]. Therefore, the mechanism by which p21 inhibits RPE cell migration may involve the PI3K/AKT signaling pathway. We aimed to verify these results in our further experiments.

To better understand the effect of saRNAs in vivo, we constructed the PVR in vivo model. Intraocular injection of ARPE-19 cells is a common method to induce vitreous proliferation response and establish the PVR animal model. Following the injection, the reaction peaks in approximately 2 weeks and tractive retinal detachment occurs at 3–4 weeks. Previous in vitro studies have demonstrated that the expression of p21 started on day 2 and peaked on day 3, lasting for 1 week. Therefore, we administered RAG1-40-53-C1 at different doses within 7 days of PVR induction. Fundus microscopy and OCT showed that the extent of proliferative membrane development and retinal detachment in the experimental group was less than that in the control group. Proliferative membrane development and retinal detachment were comparable to those in the methotrexate group. These results are consistent with those of in vitro experiments.

p21^WAF1/CIP1^ is considered to be an important CDKI with a wide range of kinase inhibitory activities that can effectively inhibit cell cycle and prevent cells from passing G1/S-phase checkpoints, thereby inhibiting cell proliferation [9]. Studies have shown that p21 inhibits the migration and proliferation of tumor cells, including those of the liver and lungs [20, 21]. PVR shares many similarities with cancer, and many approved antiproliferative drugs and cell cycle blocking drugs [22] have been tested in clinical trials for the treatment of PVR. In addition, p21 expression is low in ARPE-19 cells derived from human RPE cells, which plays an important role in PVR pathogenesis [23]. Studies have shown that p21 can inhibit RPE cell proliferation and migration in vitro and suppress PVR in vivo [8].

Together, in the present study, a novel RNA abased therapeutic approach has been tested for the treatment of PVR. By activating the expression of p21, a major negative regulator of the cell cycle and an inducer of cell senescence, using a p21 specific saRNA, we demonstrated that the proliferation and migration of human RPE cells could be suppressed with no detectable cell death or apoptosis. Conjugating a cholesterol moiety to the saRNA allowed sustainable retention in the vitreous space with ***in vivo*** pharmacological activity after intravitreal administration. Further treatment of established PVR in a rabbit model by a single intravitreal saRNA injection slowed down the progression of PVR. Findings from the present study warrantee further development of p21 saRNA for the treatment of PVR.

## Supporting information

S1 File (ZIP)
