# Supplementary figures and images for: p21CIP/WAF1 saRNA inhibits proliferative vitreoretinopathy in a rabbit model
Source: PLoS One. 2023 Feb 23;18(2):e0282063. doi: 10.1371/journal.pone.0282063 (PMC9949646; doi:10.1371/journal.pone.0282063)

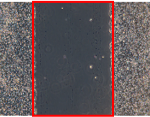

Supplement: S1 File — (ZIP) [file pone.0282063.s001.zip › ╘¡╩╝╩2╛▌/Fig3/Fig3A/ds-0h.tif]

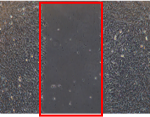

Supplement: S1 File — (ZIP) [file pone.0282063.s001.zip › ╘¡╩╝╩2╛▌/Fig3/Fig3A/ds-24h.tif]

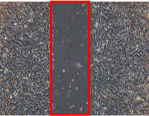

Supplement: S1 File — (ZIP) [file pone.0282063.s001.zip › ╘¡╩╝╩2╛▌/Fig3/Fig3A/ds-48h.tif]

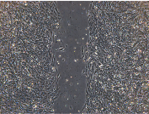

Supplement: S1 File — (ZIP) [file pone.0282063.s001.zip › ╘¡╩╝╩2╛▌/Fig3/Fig3A/ds-72h.tif]

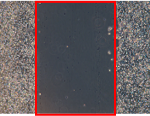

Supplement: S1 File — (ZIP) [file pone.0282063.s001.zip › ╘¡╩╝╩2╛▌/Fig3/Fig3A/mock-0h.tif]

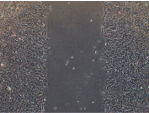

Supplement: S1 File — (ZIP) [file pone.0282063.s001.zip › ╘¡╩╝╩2╛▌/Fig3/Fig3A/mock-24h.tif]

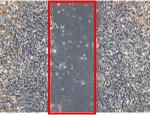

Supplement: S1 File — (ZIP) [file pone.0282063.s001.zip › ╘¡╩╝╩2╛▌/Fig3/Fig3A/mock-48h.tif]

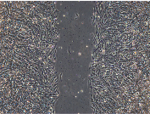

Supplement: S1 File — (ZIP) [file pone.0282063.s001.zip › ╘¡╩╝╩2╛▌/Fig3/Fig3A/mock-72h.tif]

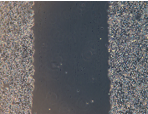

Supplement: S1 File — (ZIP) [file pone.0282063.s001.zip › ╘¡╩╝╩2╛▌/Fig3/Fig3A/NT-0h.tif]

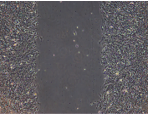

Supplement: S1 File — (ZIP) [file pone.0282063.s001.zip › ╘¡╩╝╩2╛▌/Fig3/Fig3A/NT-24h.tif]

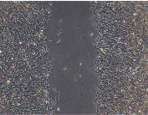

Supplement: S1 File — (ZIP) [file pone.0282063.s001.zip › ╘¡╩╝╩2╛▌/Fig3/Fig3A/NT-48h.tif]

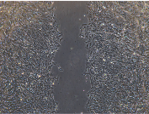

Supplement: S1 File — (ZIP) [file pone.0282063.s001.zip › ╘¡╩╝╩2╛▌/Fig3/Fig3A/NT-72h.tif]

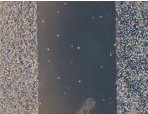

Supplement: S1 File — (ZIP) [file pone.0282063.s001.zip › ╘¡╩╝╩2╛▌/Fig3/Fig3A/RAG-0h.tif]

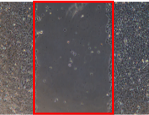

Supplement: S1 File — (ZIP) [file pone.0282063.s001.zip › ╘¡╩╝╩2╛▌/Fig3/Fig3A/RAG-24h.tif]

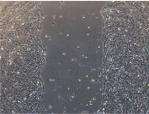

Supplement: S1 File — (ZIP) [file pone.0282063.s001.zip › ╘¡╩╝╩2╛▌/Fig3/Fig3A/RAG-48h.tif]

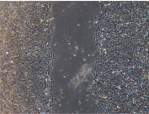

Supplement: S1 File — (ZIP) [file pone.0282063.s001.zip › ╘¡╩╝╩2╛▌/Fig3/Fig3A/RAG-72h.tif]

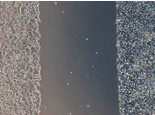

Supplement: S1 File — (ZIP) [file pone.0282063.s001.zip › ╘¡╩╝╩2╛▌/Fig3/Fig3A/TNF-DS-0h.tif]

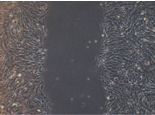

Supplement: S1 File — (ZIP) [file pone.0282063.s001.zip › ╘¡╩╝╩2╛▌/Fig3/Fig3A/TNF-DS-24h.tif]

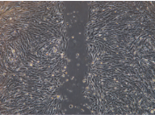

Supplement: S1 File — (ZIP) [file pone.0282063.s001.zip › ╘¡╩╝╩2╛▌/Fig3/Fig3A/TNF-DS-48h.tif]

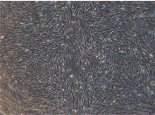

Supplement: S1 File — (ZIP) [file pone.0282063.s001.zip › ╘¡╩╝╩2╛▌/Fig3/Fig3A/TNF-DS-72h.tif]

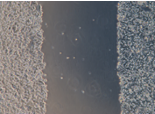

Supplement: S1 File — (ZIP) [file pone.0282063.s001.zip › ╘¡╩╝╩2╛▌/Fig3/Fig3A/TNF-MOCK-0h.tif]

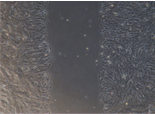

Supplement: S1 File — (ZIP) [file pone.0282063.s001.zip › ╘¡╩╝╩2╛▌/Fig3/Fig3A/TNF-MOCK-24h.tif]

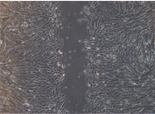

Supplement: S1 File — (ZIP) [file pone.0282063.s001.zip › ╘¡╩╝╩2╛▌/Fig3/Fig3A/TNF-MOCK-48h.tif]

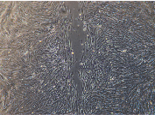

Supplement: S1 File — (ZIP) [file pone.0282063.s001.zip › ╘¡╩╝╩2╛▌/Fig3/Fig3A/TNF-MOCK-72h.tif]

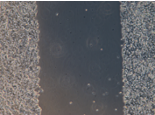

Supplement: S1 File — (ZIP) [file pone.0282063.s001.zip › ╘¡╩╝╩2╛▌/Fig3/Fig3A/TNF-NT-0h.tif]

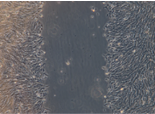

Supplement: S1 File — (ZIP) [file pone.0282063.s001.zip › ╘¡╩╝╩2╛▌/Fig3/Fig3A/TNF-NT-24h.tif]

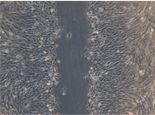

Supplement: S1 File — (ZIP) [file pone.0282063.s001.zip › ╘¡╩╝╩2╛▌/Fig3/Fig3A/TNF-NT-48h.tif]

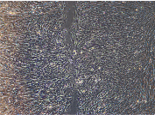

Supplement: S1 File — (ZIP) [file pone.0282063.s001.zip › ╘¡╩╝╩2╛▌/Fig3/Fig3A/TNF-NT-72h.tif]

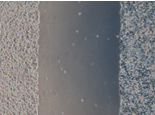

Supplement: S1 File — (ZIP) [file pone.0282063.s001.zip › ╘¡╩╝╩2╛▌/Fig3/Fig3A/TNF-RAG-0h.tif]

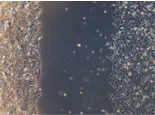

Supplement: S1 File — (ZIP) [file pone.0282063.s001.zip › ╘¡╩╝╩2╛▌/Fig3/Fig3A/TNF-RAG-24h.tif]

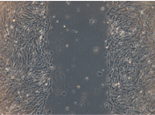

Supplement: S1 File — (ZIP) [file pone.0282063.s001.zip › ╘¡╩╝╩2╛▌/Fig3/Fig3A/TNF-RAG-48h.tif]

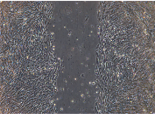

Supplement: S1 File — (ZIP) [file pone.0282063.s001.zip › ╘¡╩╝╩2╛▌/Fig3/Fig3A/TNF-RAG-72h.tif]

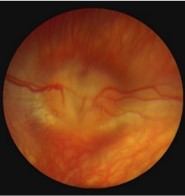

Supplement: S1 File — (ZIP) [file pone.0282063.s001.zip › ╘¡╩╝╩2╛▌/Fig6/Fig6B/═╝╞1⁄426.jpg]

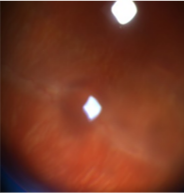

Supplement: S1 File — (ZIP) [file pone.0282063.s001.zip › ╘¡╩╝╩2╛▌/Fig6/Fig6B/═╝╞1⁄427.tif]

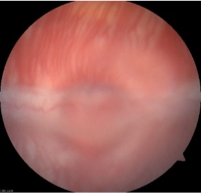

Supplement: S1 File — (ZIP) [file pone.0282063.s001.zip › ╘¡╩╝╩2╛▌/Fig6/Fig6B/═╝╞1⁄428.tif]

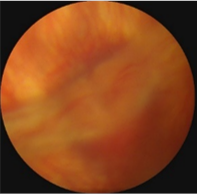

Supplement: S1 File — (ZIP) [file pone.0282063.s001.zip › ╘¡╩╝╩2╛▌/Fig6/Fig6B/═╝╞1⁄429.tif]

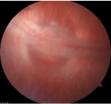

Supplement: S1 File — (ZIP) [file pone.0282063.s001.zip › ╘¡╩╝╩2╛▌/Fig6/Fig6C/D14-0.1-F.tif]

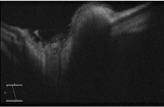

Supplement: S1 File — (ZIP) [file pone.0282063.s001.zip › ╘¡╩╝╩2╛▌/Fig6/Fig6C/D14-0.1-O.tif]

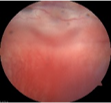

Supplement: S1 File — (ZIP) [file pone.0282063.s001.zip › ╘¡╩╝╩2╛▌/Fig6/Fig6C/D14-0.3-F.tif]

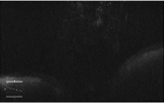

Supplement: S1 File — (ZIP) [file pone.0282063.s001.zip › ╘¡╩╝╩2╛▌/Fig6/Fig6C/D14-0.3-O.tif]

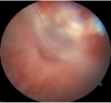

Supplement: S1 File — (ZIP) [file pone.0282063.s001.zip › ╘¡╩╝╩2╛▌/Fig6/Fig6C/D14-1-F.tif]

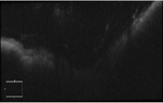

Supplement: S1 File — (ZIP) [file pone.0282063.s001.zip › ╘¡╩╝╩2╛▌/Fig6/Fig6C/D14-1-O.tif]

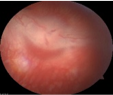

Supplement: S1 File — (ZIP) [file pone.0282063.s001.zip › ╘¡╩╝╩2╛▌/Fig6/Fig6C/D14-MTX-F.tif]

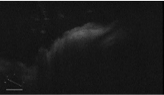

Supplement: S1 File — (ZIP) [file pone.0282063.s001.zip › ╘¡╩╝╩2╛▌/Fig6/Fig6C/D14-MTX-O.tif]

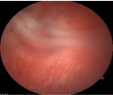

Supplement: S1 File — (ZIP) [file pone.0282063.s001.zip › ╘¡╩╝╩2╛▌/Fig6/Fig6C/D14-PBS-F.tif]

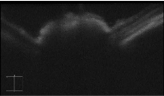

Supplement: S1 File — (ZIP) [file pone.0282063.s001.zip › ╘¡╩╝╩2╛▌/Fig6/Fig6C/D14-PBS-O.tif]

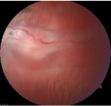

Supplement: S1 File — (ZIP) [file pone.0282063.s001.zip › ╘¡╩╝╩2╛▌/Fig6/Fig6C/D22-0.1-F.tif]

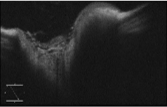

Supplement: S1 File — (ZIP) [file pone.0282063.s001.zip › ╘¡╩╝╩2╛▌/Fig6/Fig6C/D22-0.1-O.tif]

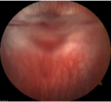

Supplement: S1 File — (ZIP) [file pone.0282063.s001.zip › ╘¡╩╝╩2╛▌/Fig6/Fig6C/D22-0.3-F.tif]

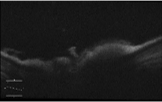

Supplement: S1 File — (ZIP) [file pone.0282063.s001.zip › ╘¡╩╝╩2╛▌/Fig6/Fig6C/D22-0.3-O.tif]

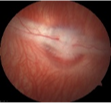

Supplement: S1 File — (ZIP) [file pone.0282063.s001.zip › ╘¡╩╝╩2╛▌/Fig6/Fig6C/D22-1-F.tif]

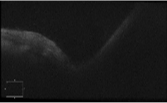

Supplement: S1 File — (ZIP) [file pone.0282063.s001.zip › ╘¡╩╝╩2╛▌/Fig6/Fig6C/D22-1-O.tif]

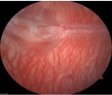

Supplement: S1 File — (ZIP) [file pone.0282063.s001.zip › ╘¡╩╝╩2╛▌/Fig6/Fig6C/D22-MTX-F.tif]

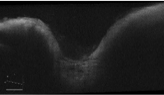

Supplement: S1 File — (ZIP) [file pone.0282063.s001.zip › ╘¡╩╝╩2╛▌/Fig6/Fig6C/D22-MTX-O.tif]

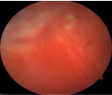

Supplement: S1 File — (ZIP) [file pone.0282063.s001.zip › ╘¡╩╝╩2╛▌/Fig6/Fig6C/D22-PBS-F.tif]

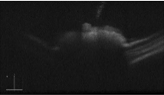

Supplement: S1 File — (ZIP) [file pone.0282063.s001.zip › ╘¡╩╝╩2╛▌/Fig6/Fig6C/D22-PBS-O.tif]
